# Diagnostic modelling and therapeutic monitoring of immune-mediated necrotizing myopathy: role of electrical myotonia

**DOI:** 10.1093/braincomms/fcaa191

**Published:** 2020-12-13

**Authors:** James D Triplett, Shahar Shelly, Guy Livne, Margherita Milone, Charles D Kassardjian, Teerin Liewluck, Cecilia Kelly, Elie Naddaf, Ruple S Laughlin, Christopher J Lamb, Devon Rubin, Elliot L Dimberg, Divanshu Dubey, John R Mills, Jay Mandrekar, Christopher J Klein

**Affiliations:** Department of Neurology, Mayo Clinic, Rochester, MN, USA; Department of Neurology, Mayo Clinic, Rochester, MN, USA; Guylivne.com, Boston, MA, USA; Department of Neurology, Mayo Clinic, Rochester, MN, USA; Division of Neurology, Department of Medicine, St. Michael’s Hospital, University of Toronto, Canada; Department of Neurology, Mayo Clinic, Rochester, MN, USA; Department of Neurology, Mayo Clinic, Rochester, MN, USA; Department of Neurology, Mayo Clinic, Rochester, MN, USA; Department of Neurology, Mayo Clinic, Rochester, MN, USA; Department of Neurology, Mayo Clinic, Rochester, MN, USA; Department of Neurology, Mayo Clinic, Rochester, MN, USA; Department of Neurology, Mayo Clinic, Rochester, MN, USA; Department of Neurology, Mayo Clinic, Rochester, MN, USA; Department of Laboratory Medicine and Pathology, Mayo Clinic, Rochester, MN, USA; Department of Laboratory Medicine and Pathology, Mayo Clinic, Rochester, MN, USA; Department of Neurology, Mayo Clinic, Rochester, MN, USA; Department of Biomedical Statistics and Informatics, Mayo Clinic, Rochester, MN, USA; Department of Neurology, Mayo Clinic, Rochester, MN, USA; Department of Laboratory Medicine and Pathology, Mayo Clinic, Rochester, MN, USA

**Keywords:** myotonia, HMGCR, SRP54, myositis, immune-mediated necrotizing myopathy

## Abstract

Delayed diagnosis of immune**-**mediated necrotizing myopathy leads to increased morbidity. Patients with the chronic course without 3-hydroxy-3-methylglutaryl-coenzyme-A reductase-IgG or signal recognition particle-IgG are often challenging to diagnose. Immunotherapy response can also be difficult to assess. We created a statistical model to assist immune**-**mediated necrotizing myopathy diagnosis. Electrical myotonia versus fibrillations were reviewed as biomarkers for immunotherapy treatment response. Identified were 119 immune**-**mediated necrotizing myopathy cases and 938 other myopathy patients. Inclusion criteria included all having electrophysiological evaluations, muscle biopsies showing inflammatory/necrotizing myopathies, comprehensively recorded neurological examinations, and creatine kinase values. Electrical myotonia was recorded in 56% (67/119) of retrospective and 67% (20/30) of our validation immune**-**mediated necrotizing myopathy cohorts, and significantly (*P* < 0.001) favoured immune**-**mediated necrotizing myopathy over other myopathies: sporadic inclusion body myositis (odds ratio = 4.78); dermatomyositis (odds ratio = 10.61); non-specific inflammatory myopathies (odds ratio = 8.46); limb-girdle muscular dystrophies (odds ratio = 5.34) or mitochondrial myopathies (odds ratio = 14.17). Electrical myotonia occurred in immune**-**mediated necrotizing myopathy seropositive (3-hydroxy-3-methylglutaryl-coenzyme-A reductase-IgG 70%, 37/53; signal recognition particle-IgG 29%, 5/17) and seronegative (51%, 25/49). Multivariate regression analysis of 20 variables identified 8 (including electrical myotonia) in combination accurately predicted immune**-**mediated necrotizing myopathy (97.1% area-under-curve). The model was validated in a separate cohort of 30 immune**-**mediated necrotizing myopathy cases. Delayed diagnosis of cases with electrical myotonia occurred in 24% (16/67, mean 8 months; range 0–194). Half (8/19) had a chronic course and were seronegative, with high model prediction (>86%) at the first visit. Inherited myopathies were commonly first suspected in them. Follow-up evaluation in patients with electrical myotonia on immunotherapy was available in 19 (median 21 months, range 2–124) which reduced from 36% (58/162) of muscles to 7% (8/121; *P* < 0.001). Reduced myotonia correlated with immunotherapy response in 64% (9/14) as well as with median creatine kinase reduction of 1779 U/l (range 401–9238, *P* < 0.001). Modelling clinical features with electrical myotonia is especially helpful in immune**-**mediated necrotizing myopathy diagnostic suspicion among chronic indolent and seronegative cases. Electrical myotonia favours immune**-**mediated necrotizing myopathy diagnosis and can serve as an adjuvant immunotherapy biomarker.

## Teaser

Delayed diagnosis of immune-mediated necrotizing myopathy leads to increased morbidity. Chronic course or antibody negative status complicates diagnosis. Immunotherapy treatment response can also be difficult to assess. Triplett *et al.* generated a statistical model to assist immune-mediated necrotizing myopathy diagnosis demonstrating the value of electrical myotonia discovery in that model and in immunotherapy monitoring.

## Graphical Abstract

**Graphical Abstract:**
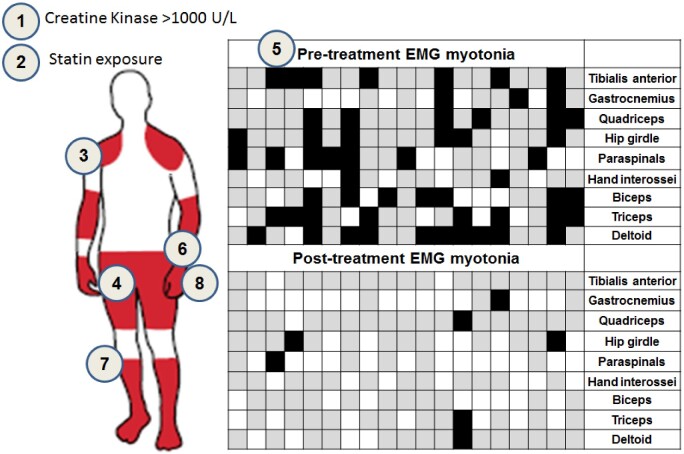

## Introduction

Immune-mediated necrotizing myopathy (IMNM) patients have progressive muscle weakness, typically with marked elevation of serum creatine kinase (CK) (Grable-Esposito *et al.*, 2010; Kassardjian *et al.*, 2015*b*). Most commonly, patients have sub-acute onsets, sometimes presenting as a medical emergency with rhabdomyolysis, loss of ambulation and respiratory failure (Allenbach *et al.*, 2014; Kassardjian *et al.*, 2015*b*). Patients with indolent chronic course have delayed diagnosis, often initially being considered to have limb-girdle muscular dystrophies (LGMD), dermatomyositis (DM) and other slower onset inflammatory myopathies (Mammen *et al.*, 2011; Allenbach *et al.*, 2014; Kassardjian *et al.*, 2015*b*; Mohassel *et al.*, 2019). Early initiation of a combination of immunotherapy results in better outcomes in IMNM (Grable-Esposito *et al.*, 2010; Suzuki *et al.*, 2012; Garcia-Rosell *et al.*, 2013; Kassardjian *et al.*, 2015*b*; Ramanathan *et al.*, 2015). Consensus guidelines now recommend first-line steroids plus IVIG, methotrexate or rituximab as initial management (Allenbach *et al.*, 2018). Muscle biopsy findings typically show scattered myofibre necrosis and regeneration without inflammation, but some patients have inflammatory infiltrates complicating diagnosis (De Bleecker *et al.*, 2015; Allenbach *et al.*, 2018). Serological testing facilitates diagnosis in 30–58% of patients either by identification of IgG-autoantibodies against 3-hydroxy-3-methylglutaryl-coenzyme-A reductase (HMGCR) or signal recognition particle (SRP54) (Allenbach *et al.*, 2014; Kassardjian *et al.*, 2015*b*; Watanabe *et al.*, 2016). SRP autoantibodies can also be identified by RNA immunoprecipitation or via cell-based immuno-assays using a 54-kDa portion of the SRP protein (Suzuki *et al.*, 2015). False-positive serology can occur especially in patients having low pretest probability of IMNM (Mammen *et al.*, 2012; Mecoli *et al.*, 2020). Currently, a clinico-sero-pathological diagnosis is the gold standard, and specific electrophysiological features have not been included to aid in the diagnosis (Allenbach *et al.*, 2018).

Needle electromyography (EMG) is an important tool in myopathy evaluations, with myopathic motor units and fibrillation potentials assisting in the determination of and optimal muscle biopsy sites (Naddaf *et al.*, 2018; Sener *et al.*, 2019). In one large series of IMNM (Kassardjian *et al.*, 2015*b*), electrical myotonia has been reported to occur in 51% (32/51) but details of the muscles affected, and outcomes with immunotherapy were not provided. Although not specific for any disorder, electrical myotonia is known to occur in myotonic dystrophies (DM1&2), non-dystrophic myotonia and less commonly in sporadic inclusion body myositis (sIBM) and toxic-metabolic myopathies (Paganoni and Amato, 2013). In the metabolic myopathy ‘Pompe’, electrical myotonia was reported in the paraspinal or tensor fascia latae muscles selectively versus generalized involvements in the other conditions (Kassardjian *et al.*, 2015*a*). The pathophysiology of electrical myotonia is not well understood, but subthreshold ion channel dysfunction in the muscle membrane is considered the primary mechanism (Metzger *et al.*, 2020).

Herein, we study whether electrical myotonia combined with other clinical features can be modelled to assist in IMNM diagnosis and serve as an adjuvant biomarker for the following immunotherapy.

## Materials and methods

The study was performed with patient written consent and institutional research board approval. This study followed the Strengthening the Reporting of Observational Studies in Epidemiology (STROBE) reporting guideline. We searched our electronic medical record (1 January 2004 to 31 December 2018) to identify all retrospective cases. We then validated the IMNM cohort with subsequent consecutive unique cases diagnosed after 1st January 2019. Inclusion criteria were as follows: age greater than 18 years, EMG, CK and comprehensive scored neuromuscular examinations all within 3 months of pathological and serological confirmatory IMNM testing. IMNM consensus definition was utilized in all patients including muscle weakness in the presence of elevated CK and muscle pathology confirming scattered necrotic myofibres without significant endomysial inflammatory exudates with or without HMGCR, SRP54 IgG positivity (Allenbach *et al.*, 2018). Other acquired inflammatory myopathy controls were also identified that included s-IBM, DM and other non-specific inflammatory myopathies (IM) (De Bleecker *et al.*, 2015; Milone, 2017; Allenbach *et al.*, 2018). The IM category consisted of cases with muscle pathology showing necrosis and regeneration with endomysial or perimysial inflammation with or without auto aggressive inflammation and clinical features not consistent with any other diagnosis including those historically labelled polymyositis. Hereditary myopathy cases with confirmatory genetic testing were also identified including LGMD, mitochondrial myopathies (MtM) and myotonic dystrophies (DM1&2). The immune-mediated and genetic disorders chosen for comparison were based on earlier reports of confusion of those disorders with IMNM and disorders where clinical and electrophysiological overlap might occur including with electrical myotonia (Lotz *et al.*, 1989; Horvath *et al.*, 2003; Kollberg *et al.*, 2005; Allenbach *et al.*, 2014; Hanisch *et al.*, 2014; Kassardjian *et al.*, 2015*b*; Kazamel *et al.*, 2016; Nojszewska *et al.*, 2018; Mohassel *et al.*, 2019).

EMG studies were performed with a disposable concentric 25–37 mm needle using standard stimulation and recording techniques (Nicolet Viking and Cadwell machines). EMG reports were reviewed for the presence of myotonic discharges and fibrillation potentials utilizing electronic data extraction from EMG reports with chart review confirmation. Myotonic discharges were defined as 20–80 Hz repetitive discharges, waxing and/or waning in amplitude and frequency. For each muscle examined during needle EMG, the presence of myotonic discharges was documented in a comment box associated with the muscle, and the presence of fibrillation potentials documented for each muscle.

All identified IMNM and a subset of the other myopathy designations were randomly selected for chart extraction of 20 clinical variables. The variables selected for review were chosen from our own experience and earlier reports of potential distinguishers between IMNM and other myopathies including statin exposure, CK elevations greater than 1000 U/l, hamstring weakness, hip flexor weakness, tibialis anterior involvement, neck weakness, absent finger flexor weakness and others (Grable-Esposito *et al.*, 2010; Barohn *et al.*, 2014; Kassardjian *et al.*, 2015*b*).

Among IMNM patients clinical examinations, CK values, HMGCR-IgG (Inova ELISA) and SRP54-IgG (EuroImmune immunoblot) autoantibody status, and EMG findings were reviewed in correlation with the initiation of immunotherapy. The examination findings of patients at diagnosis and at the time of last repeated EMG were used to measure clinical changes. Muscle strength was determined by converting to Medical Research Council (MRC) grading scale summing eight sites bilaterally (neck flexors, deltoids, biceps, wrist extensors, gluteus maximus, gluteus medius, quadriceps and ankle dorsiflexors). A normal examination corresponds to a score of 80 versus the lowest score of 0 corresponding to all 4 limbs being flail and inability to flex the neck. Clinical improvements were defined by having a reduced CK level and improved summated MRC score.

### Statistical analysis

Wilcoxon’s rank-sum test was used to assess continuous variables; Fisher’s exact test was used for binomial variables. Two-sided and *P*-values less than 0.05 were considered statistically significant for the entire cohort. Univariate and multivariate regression analysis was performed comparing the clinical variables associated with IMNM versus other myopathies.

### Data availability

The data file used to generate predicted and actual probability of IMNM prediction is provided in Supplementary Table 1 and on our online IMNM calculator (http://imnm.info/).

## Results

### Demographics of IMNM and other myopathies for electrical myotonia

Identified clinico-sero-pathologically and genetically confirmed patients with extracted EMGs totalled 1028 (IMNM = 119, DM = 157; sIBM = 245, IM = 295, MtM = 24, LGMD = 144, DM1&2 = 44). Among IMNM patients 51% (61/119) were females. Electrical myotonia was observed in 56% of IMNM patients (67/119), affecting 22% (221/995) of muscles examined. When electrical myotonia was present amongst IMNM patients, the median rate identified in muscles examined was 36% [range 10% (1 of 10 muscles) to 100% (9 of 9 muscles)]. Electrical myotonia was present in <25% of sampled muscles in 35% (24/67) patients; 25–50% of muscles in 28% (19/67) patients; 50–75% of muscles in 20% (14/67) patients and >75% of muscles in 14% (10/67) patients. Electrical myotonia was most frequently identified in the biceps (38%, 25/65), triceps (33%, 32/98), deltoid (32%, 32/99) and paraspinal muscles (27%, 20/73). In four patients, electrical myotonia was only identified in the thoracic paraspinals.

In comparison, fibrillation potentials were identified in 89% (106/119) IMNM patients and 64% (635/995) of muscles. Amongst IMNM patients with fibrillation potentials, the median frequency of identifying fibrillations was 78% [range 11% (1/9 muscles): 100% (9/9 muscles)]. Fibrillations were identified in <25% of muscles in 23% (25/106) patients, in 25–50% of muscles in 11% (12/106) patients, in 50–75% of muscles in 28% (30/106) patients and present in over 75% of muscles in 53% (56/106) patients. Fibrillations were most common in proximal muscles in the hip girdle (gluteus maximus, gluteus medius, iliopsoas and tensor fascia lata; 90%, 486/540), paraspinals (87%, 53/73), biceps (65%, 42/65), triceps (60%, 59/98), deltoid (73%, 73/99) and least commonly in distal muscles like first dorsal interosseous (50%, 49/98) and gastrocnemius (54%, 48/90).

Clinical myotonia was not reported in any IMNM patients. The median time from muscle weakness onset to our EMG among IMNM patients was 4 months (range 1 week–72 months). The median age was not significantly different among IMNM patients with electrical myotonia, 64 years (range 27–84 years) versus 57 years (range 18–87 years) in those without electrical myotonia. Approximately half (48%, 32/67) with electrical myotonia were female. The CK at initial presentation among IMNM patients was not significantly different, with an average of 7737 U/l (range 393–29 000) in patients with electrical myotonia versus 6663 U/l (537–22 080) in patients without. At the time of our first EMG, 32% (39/119) were on some type of immunotherapy, most commonly prednisone monotherapy 56% (22/39) with 17 of these patients also on a steroid-sparing immunotherapy, 8 receiving IVIG, methotrexate or rituximab. Among them, myotonic discharges were present in 55% (21/39) of those on prednisone alone and 30% (5/17) with combination immunotherapy.

Electrical myotonia was significantly more common in IMNM compared to other myopathies (inherited and acquired inflammatory) except for DM1&2, with odds ratios favouring IMNM ranging from 4.78 (sIBM) to 14.17 (MtM) (Table 1). Although statin exposure (68%, 49/72, *P* < 0.0001) and HMGCR-IgG positivity (70%, 37/53, *P* = 0.014) were most frequently associated with electrical myotonia, electrical myotonia was also seen in seronegative (51%, 25/49), SRP54-IgG positive (29%, 5/17) patients and statin naïve patients (38%, 18/47).

**Table 1:** Odds ratios of electrical myotonia in IMNM versus other myopathies

Disease category	Percent patients with myotonia	*P*-value^a^	Odds ratio estimate^b^	Lower 95% CL	Upper 95% CL
sIBM	20% (52/245)	<0.0001	4.78	2.98	7.68
DM	11% (17/157)	<0.0001	10.61	5.71	19.73
IM	13% (39/295)	<0.0001	8.46	5.16	13.87
LGMD	14% (28/144)	<0.0001	5.34	3.08	9.24
MtM	8% (2/24)	0.0005	14.17	3.19	63.02
DM1&2	88% (39/44)	<0.0001	0.17	0.06	0.45

In the total IMNM cohort myotonic discharges occurred in 56% (67/119).

a Values were significant if *P* < 0.05.

b Odds ratios < 1.0 favour other myopathies over IMNM.

### Clinical variable modelling of IMNM versus other myopathies

Univariate regression analysis of 20 clinical variables comparing 119 IMNM versus 238 other myopathy patients (LGMD = 38, DM = 44, MtM = 21, sIBM = 45, DM1&2 = 45, IM = 45) revealed electrical myotonia plus 11 other clinical features to be significantly more common and three clinical features significantly less common in IMNM (Table 2). The odds ratios (OR) > 1.0 favoured IMNM over other myopathies (range 138.30–1.01), whereas OR < 1.0 indicated that another myopathy is more likely than IMNM, e.g., finger flexor greater than finger extensor weakness (OR 0.12). Additionally, the multivariate regression analysis identified 8 of the 20 variables (including electrical myotonia) performed best in the identification of IMNM (Table 3).

**Table 2:** Clinical variables comparing IMNM versus diverse other myopathies (univariate analysis)

Independent variable	*P*-value^a^	Odds ratio favouring IMNM^b^	Lower 95% CL	Upper 95% CL
CK > 1000 U/l	<0.0001	138.30	42.22	453.11
CK > 5000 U/l	<0.0001	22.31	0.10	49.29
Statin exposure	<0.0001	17.66	9.73	32.03
Deltoid weakness	<0.0001	10.86	5.26	22.45
Gluteus maximus weakness	<0.0001	6.44	3.59	11.55
Hip flexor weakness	<0.0001	5.23	2.73	10.04
Hamstring > quadriceps weakness	<0.0001	4.61	2.65	8.02
Hamstring weakness	<0.0001	2.86	1.82	4.51
Myotonic discharges	<0.0001	2.59	1.65	4.07
Neck extensor weakness	0.0282	1.89	1.07	3.33
Neck flexor weakness	0.0109	1.78	1.14	2.78
Finger extensor weakness > finger flexor weakness	0.0405	1.74	1.02	2.97
Quadriceps weakness	0.1926	1.35	0.86	2.11
Male	0.7079	1.09	0.70	1.69
Age at presentation	0.1964	1.01	1.00	1.02
Bulbar weakness	0.8600	0.95	0.57	1.61
Ankle plantar flexor weakness	0.4077	0.73	0.35	1.53
Quadriceps > hamstring weakness	0.0106	0.40	0.20	0.81
Ankle dorsiflexor weakness	<0.0001	0.34	0.20	0.57
Finger flexor > finger extensor weakness	<0.0001	0.12	0.04	0.34

Dermatomyositis, sporadic inclusion body myositis, non-specific inflammatory myopathy, mitochondrial myopathies, limb-girdle muscular dystrophy, and myotonic dystrophy 1&2.

a Values were significant if *P* < 0.05.

b Odds ratios < 1.0 favour other myopathies over IMNM.

CL = confidence limit; IMNM = immune-mediated necrotizing myopathy.

**Table 3:** Best clinical variables in distinction of IMNM versus diverse other myopathies (multivariate regression analysis results)

Independent variable	*P*-value^a^	Odds ratio favouring IMNM	Lower 95% CL	Upper 95% CL
CK > 1000 U/l	<0.0001	86.81	20.66	364.72
Statin exposure	<0.0001	17.40	5.50	55.08
Deltoid weakness	0.0006	8.60	2.28	32.43
Gluteus maximus weakness	0.0041	3.46	1.03	11.69
Myotonic discharges	0.0105	3.80	1.36	10.32
Finger extensor weakness > finger flexor weakness	0.0061	3.25	0.95	10.84
Ankle dorsiflexor weakness	0.0170	0.24	0.08	0.78
Finger flexor > finger extensor weakness	0.0043	0.08	0.02	0.46

Dermatomyositis, sporadic inclusion body myositis, non-specific inflammatory myopathies, mitochondrial myopathies, limb-girdle muscular dystrophy, and myotonic dystrophy 1&2.

a All values were significant *P* < 0.05.

b Odds ratios < 1.0 favour other myopathies over IMNM.

CL = confidence limit; IMNM = immune-mediated necrotizing myopathy.

The multivariate regression modelling provided very high diagnostic accuracy, with 97.1% area-under-curve receiver operating characteristics distinguishing IMNM from LGMD, DM, sIBM, IM, MtM and DM1&2. Electrical myotonia and seven clinical variables in combination performed best in the distinction of other myopathies. The other variables in the best-predictive model were statin exposure, CK > 1000 U/l, deltoid weakness, gluteus maximus weakness, finger extensor greater than finger flexor weakness, absence of finger flexor greater than finger extensor weakness and absence of ankle dorsi-flexor weakness. The probability of having IMNM varied depending on the scoring of these eight variables (Supplementary Table 1). Using 75% probability as a cut-off value, the majority (76%, 90/119) of IMNM patients could be identified using our predictive algorithm, with 1.3% (3/238) of alternative myopathy (2 IM, 1 sIBM) identified using the same 75% cut-off.

### Delayed IMNM diagnosis requiring repeat muscle biopsies

Twenty-four percent (16/67) of IMNM patients with electrical myotonia underwent multiple biopsies due to diagnostic uncertainty (15 had 2 biopsies and 1 had 4 biopsies). All but three patients were seen prior to the availability of serologic testing, which ultimately was performed and positive in only 50% (8/16: HMGCR *n* = 6; SRP54 *n* = 2; seronegative *n* = 9; not available *n* = 1). Insidious chronic course occurred in 50% (8/16). Among these chronic onset cases, DM1&2 (*n* = 1), LGMD (*n* = 5), *RYR1* (*n* = 1) and MtM (*n* = 1) myopathies were thought to be the initial diagnosis but genetic testing was negative or created uncertainty with a variant of unclear significance in large gene panel testing. Eight cases had sub-acute onsets and the initial pathology suggested IM (*n* = 2), sIBM (*n* = 1) or non-diagnostic biopsy (*n* = 5) for which repeat biopsy was revisited with clinical declines. The median delay from symptom onset to IMNM diagnosis was 8.5 months (range 0–132 months). All but one patient had experienced clinical declines before IMNM diagnosis was made and combination immunotherapy commenced. In 81% (13/16) initiating recommended combination immunotherapy (Allenbach *et al.*, 2018) stabilized or improved patient course. Utilizing our multivariate prediction algorithm and data from our first clinical visit, the probability of IMNM over other myopathy diagnosis ranged from 93% to 99% (Table 4 and Supplementary Table 1).

**Table 4:** IMNM patients and calculator predictions of disease at first visit

IMNM with delayed diagnosis having multiple muscle biopsies and electrical myotonia (*n* = 16)											
Cases	Myotonic discharges	Statin exposed	Deltoid weak	Gluteus maximus weak	Finger flexor> extensor weak^a^	Finger extensor> flexors weak^a^	Ankle dorsiflexor weak	CK >1000 U/l	IMNM probability % using calculator^b^	Lower 95% CL	Upper 95% CL
1–8	+	+	+	+	−	−	−	+	99	96	100
9	+	+	+	+	−	−	−	+	99	96	100
10–11	+	+	+	−	−	+	−	+	99	96	100
12–13	+	+	+	+	−	−	+	+	96	81	98
14–15	+	+	+	−	−	−	−	+	97	82	99
16	+	+	−	+	−	−	−	+	93	74	98
**IMNM prospective validation cohort (** *n* = **30)**											
1–11	+	+	+	+	−	−	−	+	99	96	100
12–16	−	−	+	+	−	−	−	+	62	47	76
17	−	−	+	+	−	+	−	+	84	60	95
18–19	−	+	+	+	−	+	−	+	99	95	100
20–22	+	−	+	+	−	−	−	+	86	68	95
23	+	−	+	+	−	+	−	+	95	80	99
24	+	+	−	+	−	−	−	+	93	74	98
25	+	+	+	+	−	+	−	+	100	98	100
26–28	−	−	+	+	−	+	+	+	84	60	95
29	−	+	−	+	−	−	−	+	77	41	94
30	−	+	+	+	−	−	−	+	97	89	99

a If weakness not present score variable absent (−) and score present (+) if either side affected.

b Probability that clinical features favour IMNM (immune-mediated necrotizing myopathy) versus dermatomyositis, sporadic inclusion body myositis, non-specific inflammatory myopathy, mitochondrial myopathies, limb-girdle muscular dystrophy and myotonic dystrophy 1&2.

(+) = variable present; (−) = variable absent; CL = confidence limit; IMNM = immune-mediated necrotizing myopathy.

### Immunotherapy treatment and electrical myotonia

**Figure 1:**
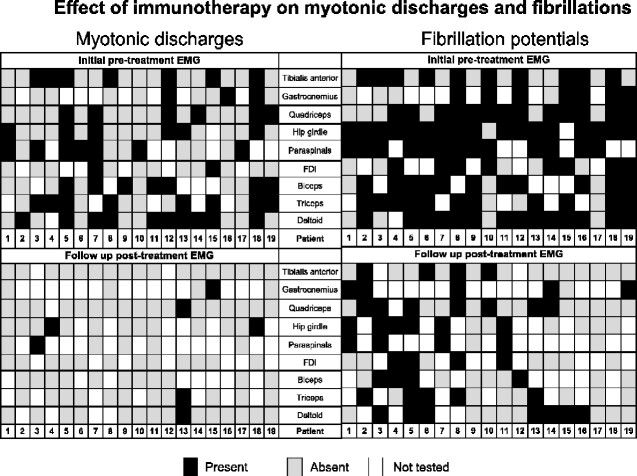
**Immunotherapy and electrical myotonia and fibrillations in IMNM.** IMNM patients (*n* = 19) undergoing serial EMGs pre-immunotherapy and at last treatment follow-up. Myotonic discharges and fibrillations reduce in frequency while on immunotherapy. On initial pre-treatment EMG Case 3 had electrical myotonia identified in both lumbar and thoracic paraspinal muscles and in Case 13 electrical myotonia was also identified in the infraspinatus muscle. On follow-up post treatment EMG Case 4 also had electrical myotonia identified in the infraspinatus muscle. Electrical myotonia resolution correlated better to treatment response than fibrillation resolution. IMNM = immune-mediated necrotizing myopathy; FDI = first dorsal interossei; Hip girdle = gluteus maximus, gluteus medius, iliopsoas or tensor fascia lata.

Thirty-eight IMNM patients underwent serial EMG and clinical evaluations, of which 19 (50%) had electrical myotonia on their initial EMG. Amongst these 19 patients, electrical myotonia was identified in 36% (58/162) of muscles sampled on the initial EMG (Figure 1). Fibrillation potentials were identified in 95% (18/19) of these patients, affecting 75% (121/162) of muscles examined and at follow-up, were present in 84% (16/19) of patients and reduced to 46% (49/121) of muscles examined (*P* < 0.001). The presence of electrical myotonia at the time of first EMG did not correlate with worse clinical severity determined by summated MRC [median 62 points (range 48–80) in patients with electrical myotonia versus 68 (range 48–80) points in those without], or with higher CK elevation [mean: 3749 U/l (range 355–9540) versus mean: 3747 U/l (range 394–11 368)].

Immunotherapy was commenced in all 19 with electrical myotonia [multidrug combination in 15: steroids (*n* = 15); IVIG (*n* = 10); azathioprine (*n* = 7)] and monotherapy in 4 [steroids (*n* = 4)]. Median EMG follow-up interval was 21 months (range 2–124 months). In these patients, post-treatment electrical myotonia was significantly less frequent, reducing from 36% (58/162) of sampled muscles to 7% (8/121) of sampled muscles (*P* < 0.001) (Figure 1). Among patients with persistent electrical myotonia, 60% (3/5) clinically worsened (all three on steroid monotherapy), 40% (2/5) were unchanged (one on steroids and azathioprine, one on steroids alone). Their median summated muscle strength score was 68 (range 28–80) at initial EMG and 64 (range 60–80) at follow-up. Their mean CK was 4430 U/l (range 355–8072) at initial EMG and 3883 U/l (range 1563–6077 U/l) at follow-up EMG. In contrast, among patients whose electrical myotonia reduced after immunotherapy, median muscle strength score improved from 66 (range 48–80) to 76 (range 56–80) at follow-up EMG, also mean CK decreased from 3506 U/l (range 465–9540 U/l) to 583 (range 47–1884 U/l). Reduced myotonia correlated with improvement or stabilization in 64% (9/14), with median CK reduction of 1779 U/l (range 401–9238, *P* < 0.001). Additionally noted is a better correlation of myotonia resolution with clinical course than with fibrillations as 84% (16/19) of the treated cohort still had fibrillations despite most having clinical improvements.

### Validation cohort

We identified 30 consecutive IMNM cases diagnosed after 1st January 2019 that were not utilized to generate our multivariate model. On EMG, 67% (20/30) had electrical myotonia: 92% (11/12) HMGCR-IgG positive, 22% (2/9) seronegative, 0% (0/4) SRP54 positive. Females accounted for 63% (19/30) with myotonic discharges in 57% (11/19) of them. Statin exposure occurred in 56% (17/30) of the entire cohort. The percent of muscles seen with electrical myotonia was 22% (53/241) and the distribution of muscles affected was similar to that seen in our larger IMNM cohort (*n* = 119). Insidious chronic course occurred in 23% (7/30) of which DM1&2 (*n* = 1), LGMD (*n* = 3), DM (*n* = 1) and IM (*n* = 2) were initially considered with median time to diagnosis 18 months (4–72 months). Utilizing our calculator, the median probability of IMNM over other myopathy diagnosis was 86% (62–100%), see Table 4. Six of 18 with myotonia on first EMG had follow-up EMGs while on recommended combination immunotherapy (prednisone plus IVIG, methotrexate or rituximab). All but one had resolutions of myotonic discharges with clinical improvements: median muscle strength score improved from 60 (range 28–60) to 76 (range 66–80) at follow-up EMG and mean CK improved 5488 U/l (range 1267–15 340) at initial EMG to 307 U/l (range 27–437 U/l) at follow-up. The one treatment-refractory patient had sustained myotonia despite mycophenolate, rituximab, prednisone and IVIG at the third follow-up EMG.

Of the 30 prospective patients, we identified two that had no myotonic discharges on initial EMG but myotonic discharges on subsequent studies. In the first case, there were clear clinical declines over 1 month on steroid monotherapy with the rise of CK by 2500 U/l. Addition of IVIG leads to stabilization. In the second case, there was equivocal worse thigh weakness which raised the possibility of type 2 fibre atrophy from high dose steroids when the CK had elevated in a borderline range, 300 U/l. However, new myotonic discharges were seen in multiple muscles (vastus, paraspinals and tensor fascia lata) previously normal and IVIG was initiated. Within 3 months clear improvements were seen (MRC 50–66 and CK value reduction of 1200 U/l).

## Discussion

The earliest clinical reports of IMNM recognized statin exposure, marked CK elevations, rapid declines, proximal muscle weakness and need for early aggressive long-term immunotherapy (Needham *et al.*, 2007; Grable-Esposito *et al.*, 2010). Our study quantifies the very high odds ratio of having CK values >1000 U/l and statin exposure in the distinction of other myopathies. Our investigation also demonstrates that combining eight clinical features including electrical myotonia can lead to a higher probability to distinguish IMNM from other myopathies having overlapping clinical and/or electrophysiological features (Lotz *et al.*, 1989; Horvath *et al.*, 2003; Kollberg *et al.*, 2005; Allenbach *et al.*, 2014; Hanisch *et al.*, 2014; Kassardjian *et al.*, 2015*b*; Kazamel *et al.*, 2016; Nojszewska *et al.*, 2018; Mohassel *et al.*, 2019). Specifically, a model utilizing electrical myotonia, statin exposure, CK > 1000 U/l, presence of deltoid, gluteus maximus, finger extensor weaknesses and absence of finger flexor and ankle dorsi-flexor weaknesses can predict IMNM at greatest accuracy by area under the curve statistical modelling.

Electrical myotonia occurred in 56% and 67% of our initial and validation IMNM cohorts respectfully, most commonly with HMGCR-IgG, but also in seronegative and SRP54-IgG positive cases. Electrical myotonia occurred in a wide variety of muscles, most frequently in the proximal upper extremity and paraspinal muscles. Paraspinal muscles should be routinely examined as these muscles oftentimes are the only muscle affected. The identification of electrical myotonia was especially helpful for those who were seronegative or had an insidious chronic course where LGMD, MtM, sIBM and other IM conditions were initially suspected and diagnosis delayed. Patients scoring >75% probability on the model should strongly be considered for an IMNM diagnosis and caution not to over-interpret inflammatory infiltrates on muscle biopsies or variants of unclear significance on genetic testing emphasized. Using the >75% cut-off, only one sIBM and two IM cases were scored of the 238 other myopathy cohort.

Utilizing the data, we have created an online calculator of the probability of IMNM (http://imnm.info/). We emphasize that a calculator based on a statistical model cannot replace sound clinical judgement; thorough history and examination and laboratory testing are still needed for a clinic-sero-pathological diagnosis of IMNM. As an example, multiple drugs and rare metabolic myopathies might produce transient rhabdomyolysis with high probability scores at one specific time (Nance and Mammen, 2015). However, in any patient presenting with sub-acute onset proximal muscle weakness, CK > 1000 U/l without a clear cause, HMGCR-IgG and SRP-IgG test should be sought and/or muscle biopsy should be considered. We hope the recognition of myotonic discharges and our calculator can reduce the delay in diagnosis we observed especially in chronic and seronegative cases as has been reported previously (Grable-Esposito *et al.*, 2010; Suzuki *et al.*, 2012; Garcia-Rosell *et al.*, 2013; Kassardjian *et al.*, 2015*b*; Ramanathan *et al.*, 2015). We also emphasize that electrical myotonia need not be present to provide accuracy in the model predictions as shown in Supplementary Table 1 where of those with 75% or greater predictions of IMNM, 44% (11/25) do not have electrical myotonia.

The exact mechanism of electrical myotonia in IMNM is unknown. Because we found statin exposure was most commonly linked to electrical myotonia, this is likely a pathological clue with membrane channel dysfunction, secondary to membrane over-expression of HMGCR or channel dysfunction following an immune response. Previous smaller studies have also provided similar inference (Meriggioli *et al.*, 2001; de Almeida *et al.*, 2008; Kassardjian *et al.*, 2015*b*). The administration of simvastatin to rabbits has been shown to produce myotonic discharges in association with elevated CK and necrotic, and/or degenerative muscle fibres on biopsy (Nakahara *et al.*, 1992). Further analysis using intracellular microelectrodes in mice showed that perfusion of normal muscles with a solution containing simvastatin or pravastatin resulted in (i) a decrease in threshold currents, (ii) prolongation of spike latency, (iii) repetitive firing and (iv) after depolarization (Sonoda *et al.*, 1994). These abnormalities were thought to represent a dysfunction of the muscle membrane, but ultimately a subthreshold channel dysfunction may explain the high observed frequency of myotonic discharges in IMNM (Metzger *et al.*, 2020). In this cohort, the resolution of electrical myotonia is typically followed by administration of combination immunotherapy and associate with better clinical outcomes than in patients with persistent electrical myotonia. Persistent myotonic discharges on subsequent EMGs may suggest ongoing disease activity and distinguish steroid myopathy from ongoing disease activity. Routine follow-up EMG is not required, but in those where therapeutic response to immunotherapy is uncertain, examining for electrical myotonia may be helpful. We saw this in one patient where steroid myopathy versus active inflammatory disease was distinguished by new myotonic discharges helping prompt escalation of treatment leading to clinical improvements. We note that electrical myotonia resolution correlated better to treatment response than resolution of fibrillations which may persist due to the presence of regenerating muscle fibres.

The limitation of this study is its retrospective nature and lack of available serological testing on all patients, with only a fraction having longitudinal follow-up. Our multivariate model is however, validated in a subset that was not used to generate the statistical model. Our multivariate model will require prospective validation in a larger cohort, but currently, this algorithm is shown helpful in considering the probability of IMNM over other myopathies. Our findings can enhance current clinico-sero-pathologic guidelines, expediting diagnosis and earlier recommended immunotherapies.

## Supplementary material

Supplementary material is available at *Brain Communications* online.

## Competing interests

Dr.  M.M. receiving research funding from a Mayo Clinic benefactor and through discretionary funding from the Department of Neurology for unrelated research projects, and an honorarium to serve as associated editor of Neurology Genetics. Dr. C.D.K. has participated in medical advisory boards for Akcea, Alexion and Takeda, and received teaching honoraria from Alexion and Sanofi Genzyme. Dr. D.D. receiving research support from the Center of Multiple Sclerosis and Autoimmune Neurology, Translational Research Innovation and Test Development Office, and Grifols Pharmaceuticals; has consulted for UCB Pharmaceuticals (all compensation for consulting activities is paid directly to Mayo Clinic); has a patent pending for KLHL11 as marker of neurologic autoimmunity; and is on the editorial board of Journal of Clinical Medicine. Dr. J.R.M. has received research support from Translational Research Innovation and Test Development Office, has patents on the use of mass spectrometry to measure monoclonal immunoglobulins, and receives royalties related to these patents from The Binding Site. Dr. C.J.K. is on the therapeutic CMTA advisory board. No authors have any conflicts of interests as related to this work.

## Funding

This work was supported by the Mayo Clinic Foundation, the Center of Individualized Medicine and the Center for MS and Autoimmune Neurology. The funders had no role in the design and conduct of the study; Collection, management, analysis or interpretation of the data; preparation, review or approval of the manuscript; or decision to submit the manuscript for publication.

## Supplementary Material

fcaa191_Supplementary_Data

## Abbreviations

DM =: dermatomyositis
DM1&2 =: myotonic dystrophies types 1 & 2
FDI =: first dorsal interossei
HMGCR =: 3-hydroxy-3-methylglutaryl-coenzyme-A reductase
IMNM =: immune-mediated necrotizing myopathy
IM =: non-specific inflammatory myopathies
IVIG =: intravenous immunoglobulin
LGMD =: limb-girdle muscular dystrophy
MtM =: mitochondrial myopathies
SRP =: signal recognition particle
sIBM =: sporadic inclusion body myositis
