# Supplementary material for: Diagnostic modelling and therapeutic monitoring of immune-mediated necrotizing myopathy: role of electrical myotonia
Source: Brain Commun. 2020 Dec 13;2(2):fcaa191. doi: 10.1093/braincomms/fcaa191 (PMC7749792; doi:10.1093/braincomms/fcaa191)
Supplement: fcaa191_Supplementary_Data [file fcaa191_supplementary_data.docx]

**Supplementary Table 1. Multivariate regression probability predictions of IMNM based on identified composite distinguishing clinical variables**

| **Multivariate model predictions** | | | | | | | | **Probability of IMNM vs other myopathies** | **Myopathy cases with clinical pattern** | | | | | | |
| --- | --- | --- | --- | --- | --- | --- | --- | --- | --- | --- | --- | --- | --- | --- | --- |
| **Myotonic discharges** | **Statin exposed** | **Deltoid weakness** | **Gluteus maximus weakness** | **Finger flexor > finger extensor weakness^a^** | **Finger extensor > finger flexor weakness^a^** | **Ankle dorsi-flexor weakness** | **Creatine kinase >1000 U/L** |  | **IMNM n=119** | **LGMD n=38** | **DM n=43** | **MtM n=21** | **sIBM n=45** | **DM 1&2 n=45** | **IM n=45** |
| + | + | + | + | - | + | - | + | 100% | 2 | 0 | 0 | 0 | 0 | 0 | 0 |
| + | + | + | + | - | - | - | + | 99% | 28 | 0 | 0 | 0 | 0 | 0 | 0 |
| + | + | + | - | - | + | - | + | 99% | 2 | 0 | 0 | 0 | 0 | 0 | 0 |
| - | + | + | + | - | + | - | + | 99% | 2 | 0 | 0 | 0 | 0 | 0 | 0 |
| + | + | + | + | - | + | + | + | 99% | 6 | 0 | 0 | 0 | 0 | 0 | 0 |
| + | + | - | + | - | + | - | + | 98% | 0 | 0 | 0 | 0 | 0 | 0 | 0 |
| + | + | + | - | - | - | - | + | 97% | 4 | 0 | 0 | 0 | 0 | 0 | 0 |
| - | + | + | + | - | - | - | + | 97% | 11 | 0 | 0 | 0 | 0 | 0 | 0 |
| - | + | + | - | - | + | - | + | 96% | 0 | 0 | 0 | 0 | 0 | 0 | 0 |
| + | + | + | + | - | - | + | + | 96% | 3 | 0 | 0 | 0 | 0 | 0 | 0 |
| + | + | + | - | - | + | + | + | 96% | 0 | 0 | 0 | 0 | 0 | 0 | 0 |
| - | + | + | + | - | + | + | + | 96% | 4 | 0 | 0 | 0 | 0 | 0 | 0 |
| + | - | + | + | - | + | - | + | 95% | 4 | 0 | 0 | 0 | 0 | 0 | 0 |
| + | + | - | + | - | - | - | + | 93% | 1 | 0 | 0 | 0 | 0 | 0 | 0 |
| + | + | - | - | - | + | - | + | 92% | 0 | 0 | 0 | 0 | 0 | 0 | 0 |
| - | + | - | + | - | + | - | + | 91% | 0 | 0 | 0 | 0 | 0 | 0 | 0 |
| + | + | - | + | - | + | + | + | 91% | 1 | 0 | 0 | 0 | 0 | 0 | 0 |
| - | + | + | - | - | - | - | + | 89% | 2 | 0 | 0 | 0 | 0 | 0 | 0 |
| + | + | + | - | - | - | + | + | 88% | 0 | 0 | 0 | 0 | 0 | 0 | 0 |
| - | + | + | + | - | - | + | + | 87% | 1 | 0 | 0 | 0 | 0 | 0 | 0 |
| - | + | + | - | - | + | + | + | 87% | 1 | 0 | 0 | 0 | 0 | 0 | 0 |
| + | - | + | + | - | - | - | + | 86% | 11 | 0 | 0 | 0 | 0 | 0 | 0 |
| + | - | + | - | - | + | - | + | 85% | 0 | 0 | 0 | 0 | 0 | 0 | 0 |
| - | - | + | + | - | + | - | + | 84% | 4 | 0 | 0 | 0 | 0 | 0 | 1 |
| + | - | + | + | - | + | + | + | 83% | 0 | 0 | 0 | 0 | 0 | 0 | 0 |
| + | + | + | + | - | + | - | - | 80% | 0 | 0 | 0 | 0 | 0 | 0 | 0 |
| + | + | - | - | - | - | - | + | 78% | 2 | 0 | 0 | 0 | 1 | 0 | 1 |
| - | + | - | + | - | - | - | + | 77% | 1 | 0 | 0 | 0 | 0 | 0 | 0 |
| - | + | - | - | - | + | - | + | 76% | 0 | 0 | 0 | 0 | 0 | 0 | 0 |
| + | + | - | + | - | - | + | + | 75% | 0 | 0 | 0 | 0 | 0 | 0 | 0 |
| a. If no weakness should score as absent (-) and if present either side (+).  Key: (+) = variable present (-) = variable absent, IMNM=immune mediated necrotizing myopathy, LGMD=limb girdle muscular dystrophies, DM1&2=myotonic dystrophy types 1 and 2, MtM=mitochondrial myopathies, sIBM= sporadic inclusion body myositis, DM=dermatomyositis, IM=inflammatory myopathy non specific | | | | | | | |  | n=90 | n=0 | n=0 | n=0 | n=1 | n=0 | n=2 |
